# Supplementary material for: Influence of Light on Particulate Organic Matter Utilization by Attached and Free-Living Marine Bacteria
Source: Front Microbiol. 2019 Jun 4;10:1204. doi: 10.3389/fmicb.2019.01204 (PMC6558058; doi:10.3389/fmicb.2019.01204)
Supplement: Supplementary file 2 [file Data_Sheet_1.pdf]

Supplementary Figures

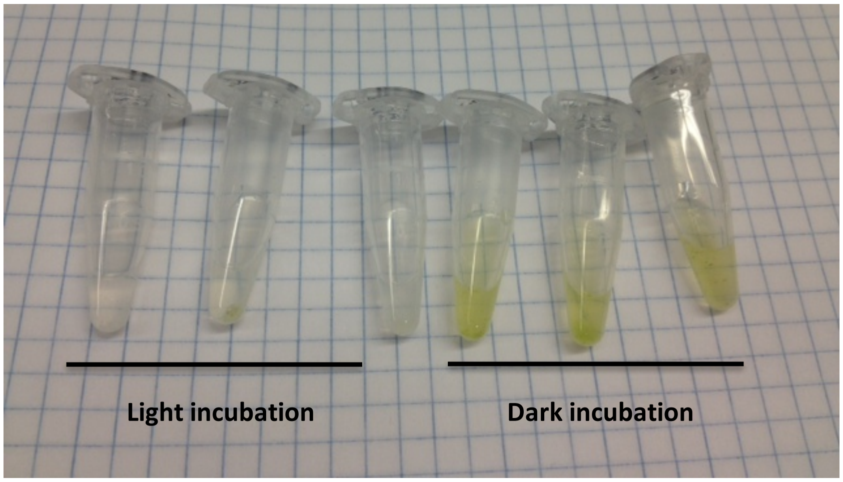

Figure S1. Visual appearance of the biomass in the experiment bottles after 72-hours incubation in continuous light (left) and dark (right). 5 ml samples from each incubation bottle were fixed with 10% formalin (3.7% formaldehyde), concentrated by centrifugation (10,000 rpm) and resuspended in 50 µl PBS.

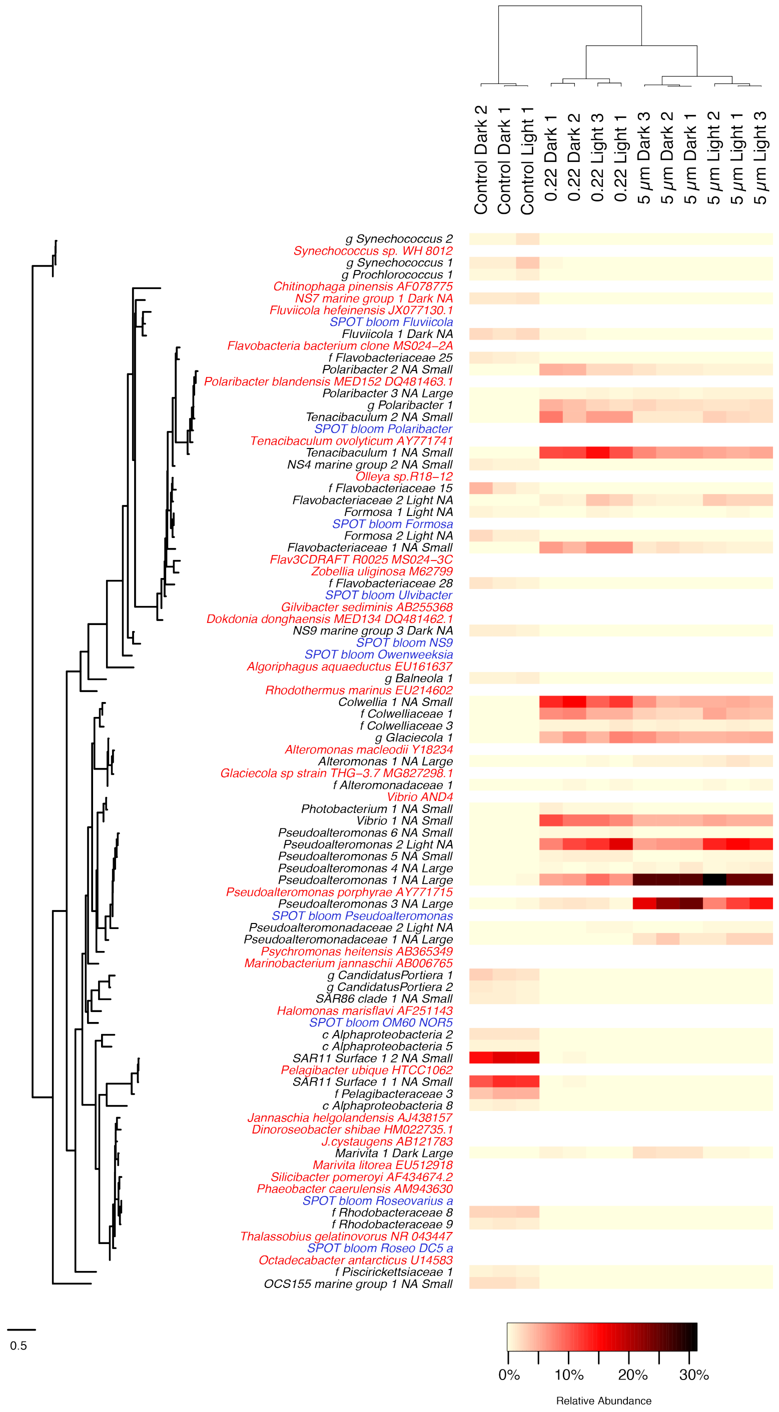

Figure S2. Heatmap that shows the 25 most relatively abundant OTUs, on average, in experimental treatments and controls. The two sets were mutually exclusive. Taxa identifiers in black are OTUs from the current study, in red are genomic reference sequences and in blue are OTUs from a natural phytoplankton bloom at the San Pedro Ocean Time-Series (SPOT) location.

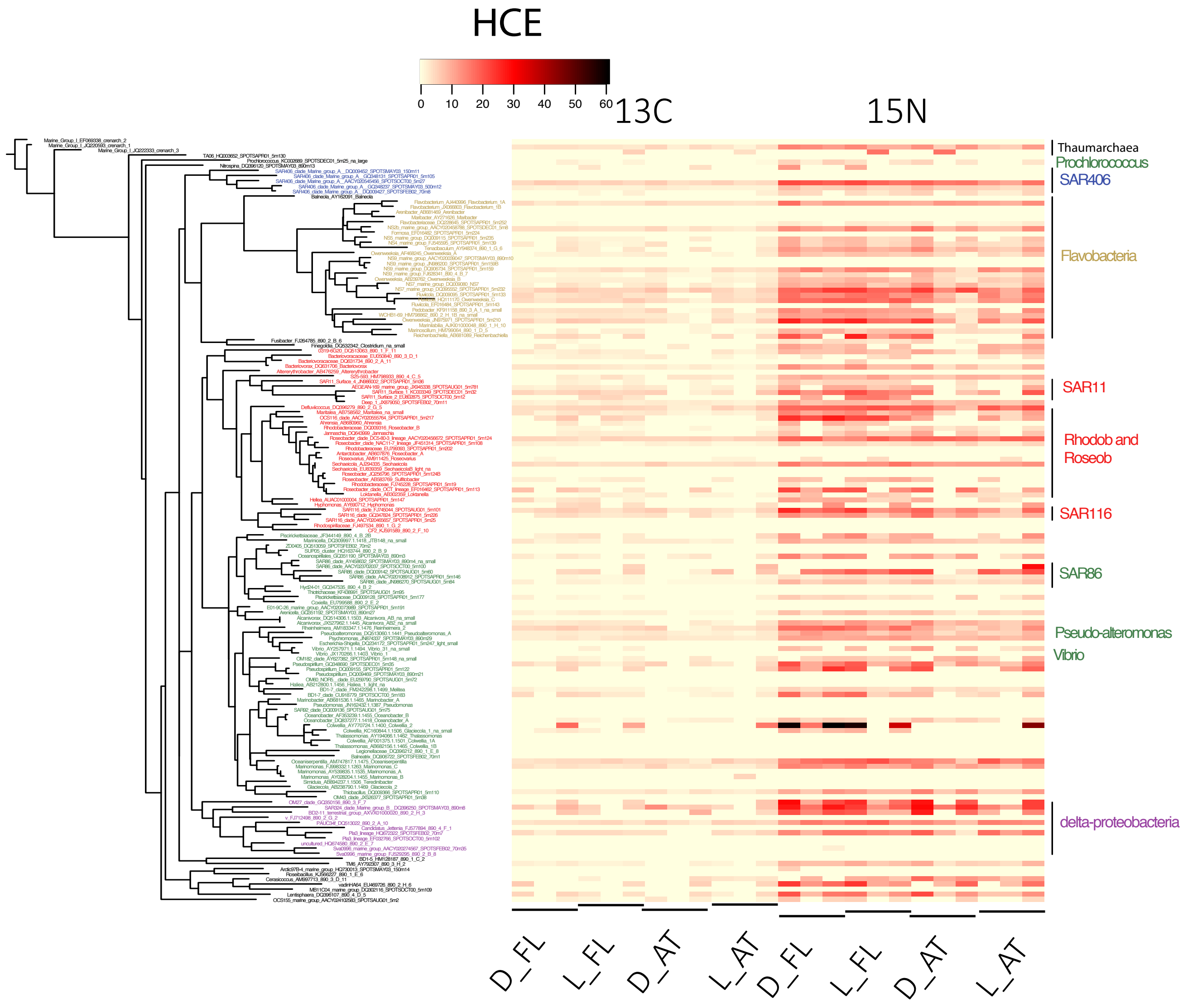

Figure S3. 16S rRNA gene phylogenetic tree of the 156 operational taxonomic units (OTUs) targeted by the Chip-SIP microarray. The heat map shows relative isotope incorporation calculated from both the ^{13}C and ^{15}N incorporation data from the 12 samples (D_FL = dark free-living, L_FL = light free-living, D_AT = dark attached and L_AT = light attached). OTU colors correspond to major bacterial phyla: Deferribacters in blue, Bacteroidetes in yellow, Alphaproteobacteria in red, Gammaproteobacteria in green, Deltaproteobacteria in purple.

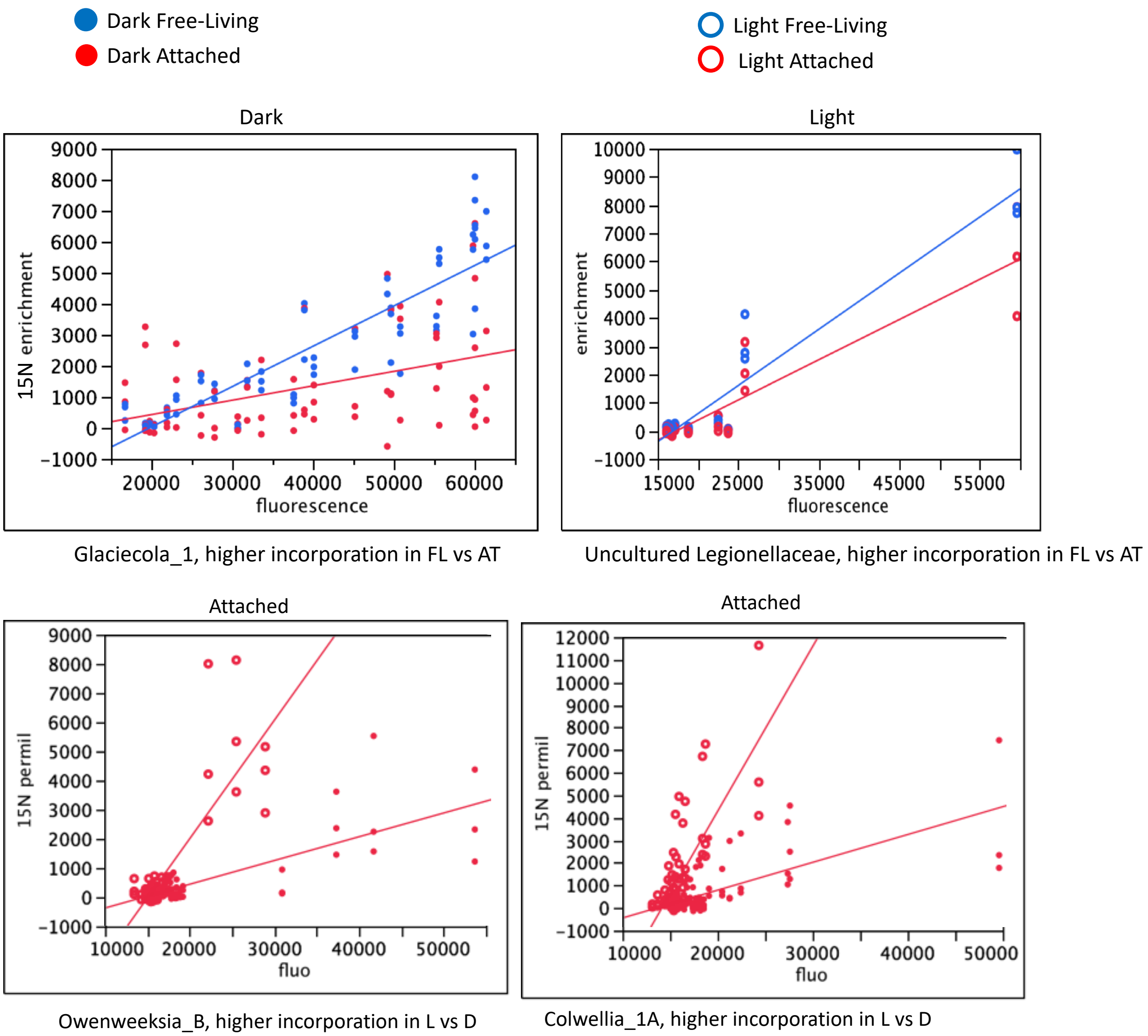

Figure S4. Analysis of covariance (ANCOVA) of two OTUs showing statistically significantly different treatment effects on isotope incorporation.

Table S1 (separate excel file). List of probe sequences used for the Chip-SIP analyses, including taxa, probe names (each probe with a unique name), probe sequence on the Chip (reverse complement of the RNA sequence targeted), and taxonomy based on SILVA.

Table S2. Classification of rRNA gene sequences retrieved by iTAG sequencing. The primers used (515F and 926R) amplify 16S rRNA gene sequences of Bacteria, Archaea and chloroplasts (Parada et al., 2015; Needham and Fuhrman, 2015), as well as eukaryotic 18S rRNA. This allows to determine the relative ratios of 16S rRNA genes of Bacteria and Archaea to eukaryotic 18S rRNA.

	Bacterial + Archaeal 16S		Eukaryotic 18S		Chloroplast 16S	
Sample	Total sequences	Percent	Total sequences	Percent	Total sequences	Percent
0.2 µm Dark 1	20,324	99.23	36	0.18	122	0.60
0.2 µm Dark 2	19,415	99.60	10	0.05	67	0.34
0.2 µm Light 1	18,993	99.31	24	0.13	108	0.56
0.2 µm Light 3	13,998	98.80	30	0.21	140	0.99
5.0 µm Dark 1	19,943	99.70	10	0.05	51	0.25
5.0 µm Dark 2	10,058	99.38	9	0.09	54	0.53
5.0 µm Dark 3	13,189	99.48	10	0.08	59	0.45
5.0 µm Light 1	24,460	99.41	30	0.12	115	0.47
5.0 µm Light 2	23,811	99.59	17	0.07	82	0.34
5.0 µm Light 3	30,955	99.44	30	0.10	143	0.46
